# An Immortal Life

**Published:** 1872-02

**Authors:** 


### AN IMMORTAL LIFE.

The works of the good come after them ; the influences of their lives widen and extend, like the waves caused by a pebble cast into the ocean, and they travel on for countless ages.

Without the influence of the clergy in every civilized nation, human rule would degenerate into anarchy, violence, and bloodshed. Without the preaching of the Gospel, and the institution of the Sabbath day, our own country would be a ruin in half a century; without the restraining influence of preaching and the Sabbath, the Commune dominates in infidel France, and the Ku-Klux rules in Free America.

There are five hundred young men in the Presbyterian Church alone, and five times the number in other denominations, healthy, talented, pious, who want to be educated for the ministry, but who have not the means to clothe themselves, pay their board, purchase books, and meet tuition fees for the five years necessary, on an average, to learn what is wanted to fit them scholastically for the position. Suppose a man or woman lays aside five thousand dollars, the interest of which should be appropriated permanently for the support at college of one of these young men, and his successors. Thereafter, then, such a person will be sending a minister into the field every few years for ages to come, and by and by there will be six or eight or ten laborers at a time, in the great work of holding up the morals of the world, and all that through the instrumentality of one person's gift of five thousand dollars. In view of a fact like this, well may it be worth the aim and effort of the lifetime of any man to be able to lay by such a sum for such a purpose. Thus his good deeds will be living after him. For ages to come, he lives in them, and although dead, yet speaketh. His work never dies, he is immortal. Each clergyman, in the course of a life-time, persuades from five to fifty, or five hundred, "to become a Christian," at least to live sober, temperate, and industrious lives; they at least are saved from vice and crime and disease and premature death, and in their stead are promoted the virtues and habits which elevate and purify and refine. Some readers of this page can lay by the sum named, called "A scholarship," and not miss it; yes, could establish a dozen scholarships and not miss it really. Others may not have it to spare to-day, but with a little effort and self-denial could do it within the year. Others could make it before they die, and thus become immortal too.

